# DNA Virome in Cardiac Tissue from Green Sea Turtles (*Chelonia mydas*) with Myocarditis

**DOI:** 10.3390/v16071053

**Published:** 2024-06-29

**Authors:** Christabel Hannon, Subir Sarker, Willy W. Suen, Helle Bielefeldt-Ohmann

**Affiliations:** 1 School of Veterinary Science, The University of Queensland, Gatton, QLD 4343, Australia; 2 Biomedical Sciences and Molecular Biology, College of Public Health, Medical and Veterinary Sciences, James Cook University, Townsville, QLD 4811, Australia; subir.sarker@jcu.edu.au; 3 Australian Centre for Disease Preparedness, CSIRO, Geelong, VIC 3220, Australia; 4 School of Chemistry & Molecular Biosciences, The University of Queensland, St. Lucia, QLD 4072, Australia; 5 Australian Infectious Diseases Centre, The University of Queensland, St. Lucia, QLD 4072, Australia

**Keywords:** sea turtle, *Chelonia mydas*, myocarditis, virome, circovirus

## Abstract

As part of a sea turtle health monitoring program on the central east coast of Queensland, Australia, stranded and sick green sea turtles (*Chelonia mydas*) were subjected to necropsy and histopathology. A subset of these turtles had myocarditis of varying severity, which could not be attributed to parasitism by spirorchid flukes or bacterial infections. We, therefore, undertook an investigation to determine whether virus infections might be part of the pathogenesis. Deep sequencing revealed abundant DNA virus contigs in the heart tissue, of which CRESS and circoviruses appeared to be the most consistently present. Further analysis revealed the homology of some of the circoviruses to the beak and feather disease virus. While a causative link to myocarditis could not be established, the presence of these viruses may play a contributing role by affecting the immune system and overall health of animals exposed to pollutants, higher water temperatures, and decreasing nutrition.

## 1. Introduction

While cardiovascular lesions are relatively common findings in sick, injured, and/or stranded sea turtles, there are few systematic investigations of the causes of such lesions, and most are ascribed to parasitism by spirorchid flukes, including *Haemoxenicon* sp., *Laeredius* sp., *Carettacola* sp., *Neospirorchis* sp., *Amphiorcoris* sp., and *Hapalotrema* sp. [1,2,3,4,5]. *Chlamydia* sp. has been reported to cause necrotizing bacterial myocarditis in juvenile green sea turtles (*Chelonia mydas*) in a mariculture setting [6]. Systemic chlamydiosis with myocarditis was also reported in another farmed reptile species, American alligators (*Alligator mississippiensis*) [7], and, hence, stress may be an important factor in this particular infection. In contrast, viral causes of endo and myocarditis appear not to have been previously investigated in sea turtles or more generally in reptiles, despite the acknowledged importance of cardiotropic viruses in mammals, with parvovirus B19, adenoviruses, and human herpes virus-6 being the most common agents in humans with acute myocarditis [8]. However, even in humans, the etiology of myocarditis is often poorly investigated [9], while the contention is that virus-triggered immune-mediated reactions are the principal cause of cardiomyocyte injury rather than actual direct virus-mediated cell injury [8,9,10]. Hence, the failure to pursue this in other species with less available diagnostic resources may not be surprising, despite the potential importance for the management and conservation of threatened species.

During the post mortem examination of green sea turtles (GSTs) stranded on the Central Queensland Coast, specifically within Port Curtis from 2017 to 2020, and animals that had been in veterinary care but were unresponsive to treatment and, therefore, terminated during the same period, a subset of GSTs were found to have mild to very severe myocarditis, endocarditis, or both. Only some of the animals had a mostly mild spirorchiid infestation, and signs of bacterial infection were not found. This prompted us to investigate possible viral infections in these animals.

## 2. Materials and Methods

### 2.1. Animal Handling and Tissue Sampling

**Figure 1:**
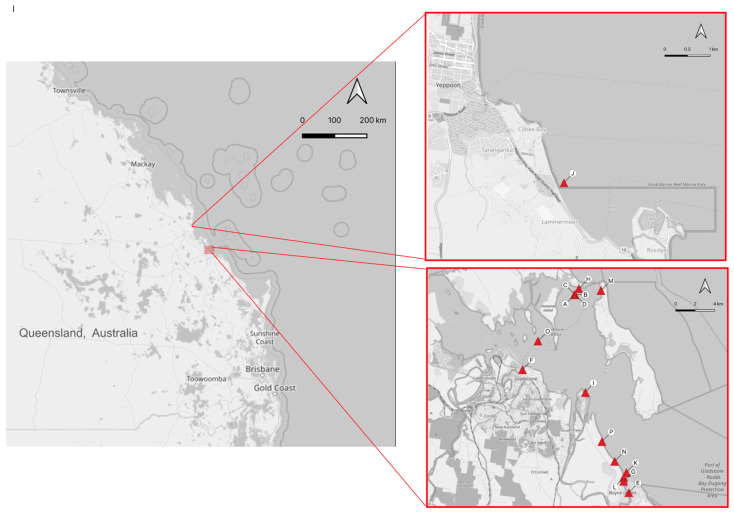
Map of Queensland, Australia, with GPS plotting of the 16 green sea turtles at Port Curtis and Yeppoo that underwent both a gross post mortem examination and histopathological assessment. Letters A–P are indicators for locations.

From September 2017 to November 2020, 22 deceased green sea turtles underwent post mortem examination and, where possible, histopathological examination. The post mortem survey was conducted as part of the Gladstone Ports Corporation Ecosystem Research and Monitoring Program (ERMP) and the Long Term Turtle Management Plan (LTTMP) efforts to determine the major causes of morbidity and mortality in green sea turtles in Gladstone Harbour, QLD (−23.76624, 151.30533) (Figure 1).

The turtles underwent necropsy according to previously established guidelines [11]. Morphometric data were collected for all turtles and are summarized in Table S1. A thorough, gross examination of all organ systems was then conducted. Where tissue autolysis was not too advanced to preclude histological interpretation, this was followed by sampling of tissues and preservation in 10% neutral-buffered formaldehyde for transportation and storage. The GPS coordinates denoting the site of the turtle discovery were recorded and mapped using QGIS [12].

All turtles died spontaneously of natural causes or were humanely euthanized for welfare reasons by a registered veterinarian. Turtle handling, sampling, and research activities were undertaken in accordance with the standard practices approved under the DAFF Animal Experimentation Ethics Committee: Queensland Turtle Conservation Project SA 2018-11-660, 661, 662, 663, and 664. The studies also received approval from the University of Queensland Animal Ethics Committee (permit no. AE43201).

### 2.2. Histology

A total of 16 of the 22 necropsied animals had tissues fixed in 10% neutral buffered formalin. The tissues were processed using standard methods and embedded in paraffin wax. Bone samples were decalcified with 8% formic acid prior to processing. Sections were cut at 5 µm and stained with hematoxylin and eosin (HE). Special stains included Gram stain to demonstrate bacteria, Grocott’s methenamine silver (GMS) and periodic acid-Schiff (PAS) stains to demonstrate fungi, and Ziehl-Neelsen stain to demonstrate *Mycobacterium* spp. All histology slides were interpreted by specialist veterinary pathologists. Counts of spirorchiid ova and adult trematodes were performed on cardiac ventricle and atrial tissues by scanning all available sections at 10× objective and are presented as a range in Table S1.

### 2.3. Nucleic Acid Recovery

Nucleic acids were isolated from the hearts of 12 animals with varying degrees of cardiac pathology. The tissues had been fixed in 10% neutral-buffered formaldehyde for periods ranging from a few weeks to several months, followed by storage in 70% ethanol until processing for nucleic acid purification 0.5–2.5 years later. The tissues were aseptically resuspended and homogenized vigorously in sterile phosphate-buffered saline (PBS) and centrifuged. The clarified supernatant was filtered before being ultracentrifuged at 178,000× *g* for 1 h (30 psi for 1 h) at 4 °C using a Hitachi Ultracentrifuge CP100NX. The supernatant was discarded, and the pellet was suspended in 130 µL of sterile PBS. The filtrates were nuclease-treated using benzonase nuclease and micrococcal nuclease. The nuclease reaction was stopped by adding EDTA. Viral nucleic acids were extracted using the QIAamp Viral RNA Mini kit (Qiagen, Valencia, CA, USA) without adding any carrier RNA, which allows the simultaneous extraction of both viral DNA and RNA. The quantity and quality of the isolated nucleic acids were determined using a Qubit 4 Fluorometer (Invitrogen, Mt Waverley VIC, Australia) and an Agilent Tape Station (Agilent Technologies, Mulgrave, VIC, Australia) by the Genomic Platform at La Trobe University.

Before library construction, extracted nucleic acids were subjected to cDNA synthesis, and amplification was carried out using the Whole Transcriptome Amplification kit (WTA2, Sigma-Aldrich, North Ryde, NSW, Australia) as per manufacturer instructions. Amplified PCR products were purified using the Wizard^®^ SV Gel and PCR Clean-Up kit (Promega, Madison, WI, USA). The quantity and quality of the purified product were checked using a Qubit dsDNA high-sensitivity assay kit with a Qubit Fluorometer v4.0 (Thermo Fisher Scientific, Waltham, MA, USA). The library construction was adapted using the Illumina Library Prep (Illumina, San Diego, CA, USA) as per kit instructions. The quality and quantity of the prepared library were assessed by the methods mentioned above. The final pooled library was further assessed before sequencing by the sequencing facility, and sequencing of the pooled library was performed with read lengths of 150 bp paired-end on the Illumina platform at the Australian Genome Research Facility, Melbourne.

### 2.4. Bioinformatics

Raw sequence reads from high-throughput sequencing were used to obtain complete genome sequences of viruses of interest as per a protocol described previously [13,14] using the CLC Genomics Workbench (version 9.5.4), Geneious (version 10.2.2), and the LIMS-HPC system (a High-Performance Computer specialized for genomics research at La Trobe University). Briefly, all raw sequencing reads were evaluated for quality and trimmed to remove the adapter sequences.

Further analysis of DNA viruses was performed by bioinformaticians at the Genomics Research Platform, La Trobe University, VIC. The following three strategies for analysis were applied: (1) abundance analysis based on mapping of sequence reads from the list generated as described above; (2) filtering of viral reads by excluding reads belonging to the host genome, fungi, and bacteria, followed by a combination of de novo assembly and local blast; and (3) abundance analysis based on mapping of the sequence reads to all viral genomes deposited in NCBI. The latter two approaches were subsequently abandoned because of the high number of reads in each sample, of which most appeared to be environmental DNA (eDNA) and only a small fraction (<1% in several samples) were from viruses. That, combined with the fact that the number of viruses in the NCBI database exceeds three million, meant that even after two weeks of computing on a high-capacity system, no output had been generated. Consequently, only data from the first approach are reported here. Furthermore, the RNA quality of the extracted nucleic acids was suboptimal, and, hence, only results for DNA viruses could be reliably analyzed.

### 2.5. Phylogenetic Analysis

For phylogenetic analyses, representative viral genomes or gene sequences were downloaded from GenBank, and virus-specific trees were constructed using the CLC Genomics Workbench (version 9.5.4) and Geneious software (version 23.1.1, Biomatters, Auckland, New Zealand). Selected nucleotide sequences were aligned using the MAFFT L-INS-i algorithm implemented in Geneious (version 7.388) [15]. To determine the best-fit model for constructing phylogenetic trees, a model test was performed using the CLC Genomics Workbench (version 9.5.4) with default parameters. This test favored a general-time-reversible model with gamma distribution rate variation and a proportion of invariable sites (GTR+G+I). Phylogenetic analyses were then performed using the GTR and WAG substitution models, respectively, with 1000 bootstrap replicates for support in Geneious software (version 23.1.1).

### 2.6. Transmission Electron Microscopy (TEM)

Formalin-fixed tissue samples were initially sectioned to generate two halves, with one destined for routine histology processing and HE staining and the other for transmission electron microscopy (TEM). Once a suitable region was identified on histopathology, the corresponding region on the opposite half of the fixed sample for TEM was excised as an approximately 1 mm^3^ cube. The samples were then conventionally processed into Spurrs resin (ProSciTech, Kirwan, QLD, Australia), sectioned, and stained, as previously described in [16]. All electron micrographs were acquired using a JEOL JEM-1400 120 KV electron microscope and Gatan Ultrascan 1000 camera (Gatan, Pleasanton, CA, USA).

## 3. Results

### 3.1. Gross Pathology and Histopathology Findings

**Figure 2:**
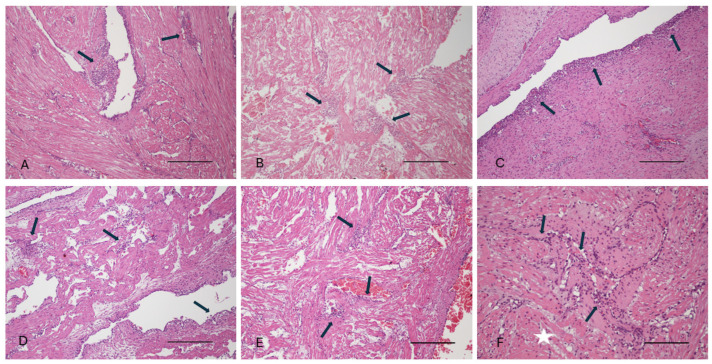
Examples of endo- and myocarditis in six of the investigated GSTs (**A**–**F**). Arrows point to more intense infiltration of lymphocytes and macrophages sub-endothelially in the endocardium and interstitially in the myocardium. White star in (**F**): area of cardiomyocyte degeneration. Scale bars in (**A**–**E**): 600 µm; scale bar in (**F**): 300 µm.

**Figure 3:**
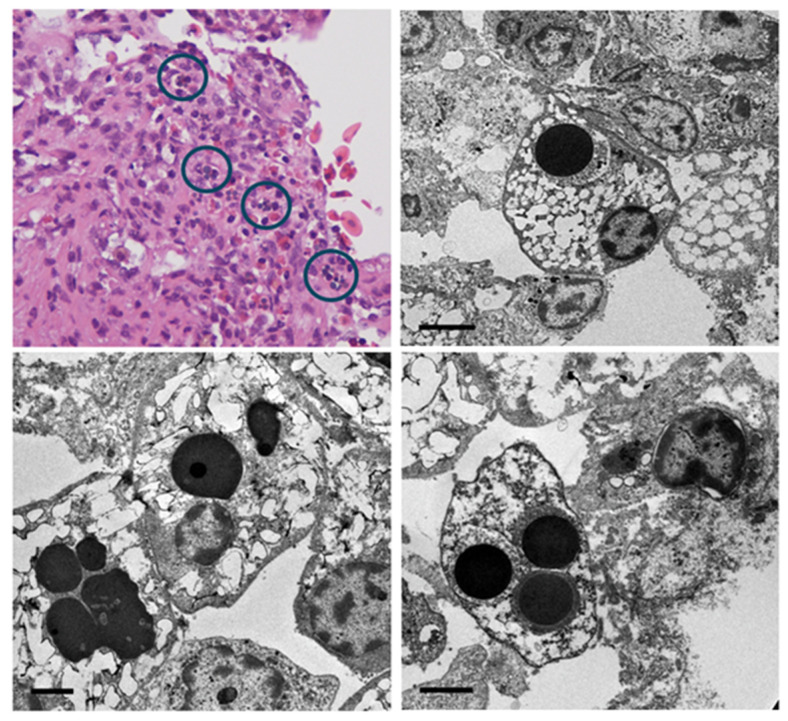
Light microscopic (top left) and TEM appearance of cytoplasmic inclusions in leukocytes in the subendothelial stroma of a green sea turtle with endo- and myocarditis. Top left panel: examples of inclusion-containing cells in the endocardium are circled. Top right panel: scale bar = 4 µm. Bottom panels: scale bars = 2 µm.

A summary of the biodata and histological results obtained from the 16 turtles that underwent both a gross post mortem examination and histological examination is summarized in Table S1. Histologically, the inflammatory leukocyte infiltrates were lymphoplasmacytic and histiocytic (Figure 2), with a few presenting as a more granulomatous inflammatory reaction, but no case of suppurative (granulocytic) inflammation was noted, making a bacterial etiology highly unlikely. This was supported by negative findings in Gram-stained sections. Nor were fungal organisms detected in either HE, GMS, or PAS-stained sections. The presentation, therefore, led to a presumptive diagnosis of virus-associated endo- and/or myocarditis. However, only in a few cases were intracytoplasmic inclusion bodies noted in inflammatory or subendothelial cells of the endocardium (Figure 3), and these were present in poorly preserved specimens and, therefore, might be caused by post mortem intracellular protein aggregation. This contention was supported by TEM examination of cardiac samples, which showed that histiocytic cells in the endocardium contained electron-dense spherical bodies with no discernible viral particles within or adjacent to them. The particles were tentatively identified as remnants of erythrocytes (Figure 3).

Nevertheless, to further investigate a potential viral etiology of the cardiac lesions, samples were subjected to nucleic acid extraction and deep sequencing with the aim of characterizing the cardiac virome. Likely due to the suboptimal preservation status of most samples prior to fixation in formaldehyde and lengthy fixation time, RNA of sufficient quality could not be recovered, and, hence, only the DNA virome could be characterized in these samples.

### 3.2. Nucleic Acid Recovery and Bioinformatics

**Figure 4:**
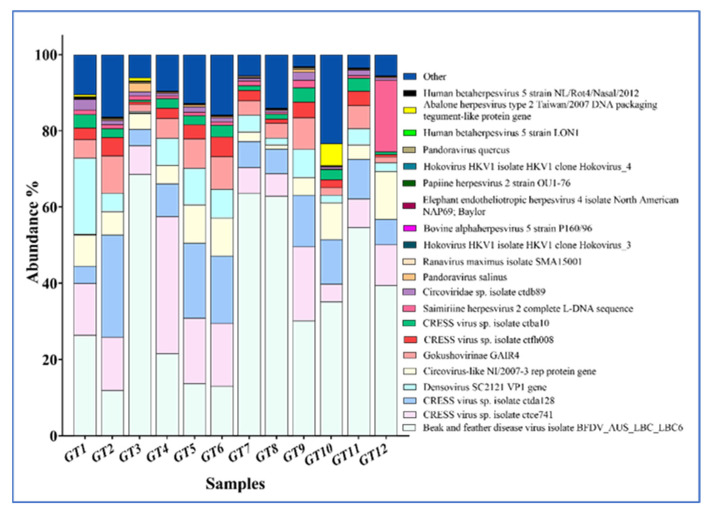
Relative abundance of DNA viruses in the heart tissue of 12 green sea turtles (refer to Table S1).

A total of 138 viruses were detected in the 12 samples analyzed based on FKPM (fragments per kilobase of exon per million mapped), with 102 viruses detected in at least one sample (GT1-GT12 in Table S1). Figure 4 shows the relative frequency of the most abundant DNA viruses in the samples. Amongst those are two Pandoraviruses, which most likely are contaminants originating from the environment, as these viruses are known to infect free-living amoeba [17]. Notably, circo- and other CRESS viruses were among the most abundant viruses in all samples, regardless of the severity of the myo-/endocarditis (Figure 4).

**Figure 5:**
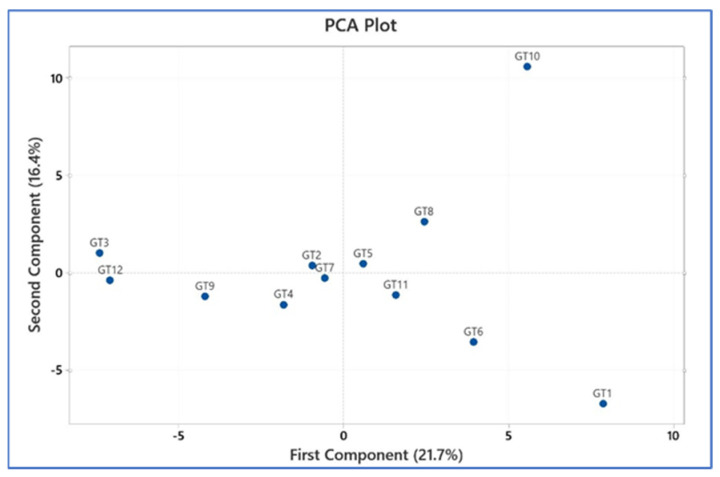
Principal component analysis (PCA) of the virome profiles in order to uncover potential etiological virus constellations.

Principal component analysis (PCA) showed that the virome profiles of GT3 and GT12 were very similar, while the virome profiles of G1 and GT10 differed substantially from all other samples (Figure 5). The remainder of the animals had relatively similar virome profiles.

**Figure 6:**
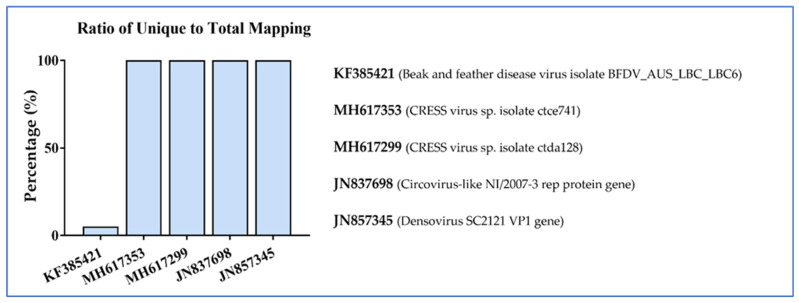
Examples of the level of genome coverage for several DNA viruses with relatively small genomes.

The fraction of viral genomes that were covered by sequence reads in each sample was calculated. The coverage varied depending on the virus and sample, ranging from 0.2% of the viral genome to 100% of the viral genome, with many of the smallest genomes (denso-, circo-, and CRESS viruses) often achieving 100% coverage (examples shown in Figure 6).

### 3.3. Phylogenetic Analysis

**Figure 7:**
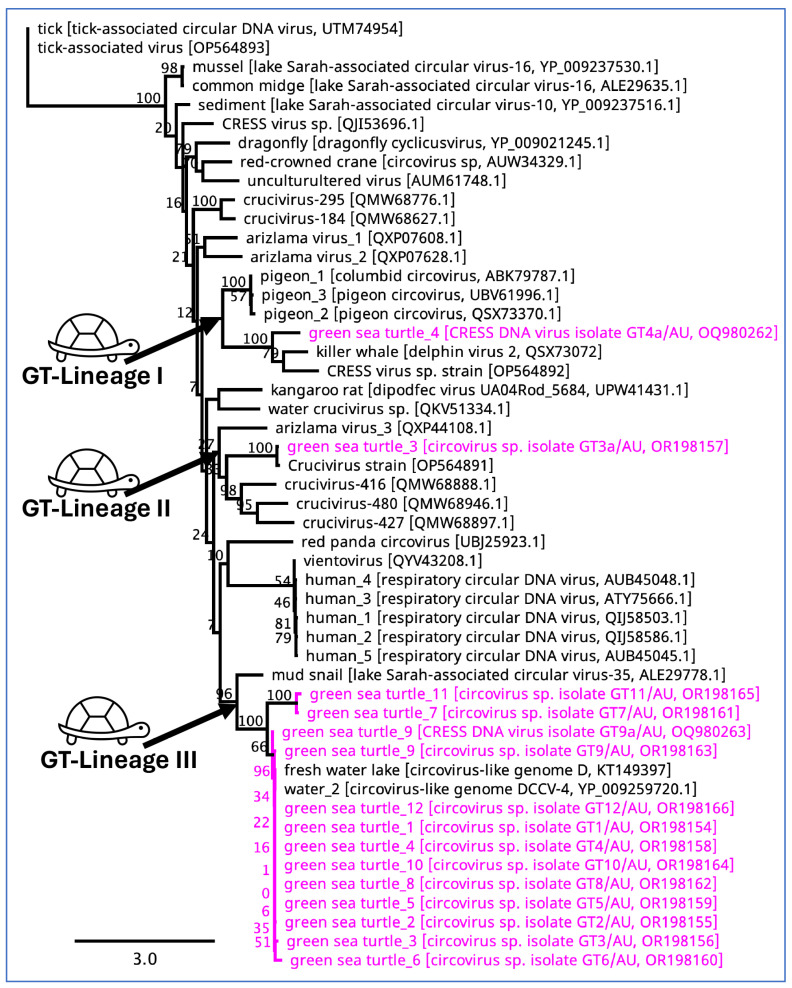
Phylogenetic tree of circoviruses with placement of the circoviruses detected in the green sea turtles of this study. The numbers on the left show bootstrap values as percentages, and the labels at branch tips refer to the original sampling host, followed by the circovirus name and GenBank accession numbers in parentheses. The sequences that correspond to the circoviruses detected in this study are shown in pink.

Circoviruses sequenced in this study were clustered into three distinctive lineages in the resulting Maximum Likelihood (ML) tree (Figure 7). Lineage I, consisting of one circular DNA virus from turtle 4 (GenBank accession number: OQ980262), showed strong bootstrap support with circoviruses sequenced from the kidney tissue and blood of killer whales and pigeons, respectively. In contrast, one circovirus sequenced from GT3 (GenBank accession number: OR198157) was shown to be evolutionarily linked with circoviruses detected in birds and water from Australia, New Zealand, and the USA (Lineage II). Furthermore, 13 circoviruses sequenced from other GSTs demonstrated strong bootstrap support (96–100%) with various circular DNA viruses sequenced from mud snails and freshwater in New Zealand and China (Lineage III). Additionally, another well-characterized circovirus in birds, the beak and feather disease virus (BFDV), was detected in seven different GSTs. However, these did not show any obvious close evolutionary relationship with previously sequenced BFDV (Supplementary Figure S1).

## 4. Discussion

During the characterization of pathologies of stranded GSTs, a conspicuous finding in 11 of 16 animals was lymphoplasmacytic, histiocytic, or granulomatous myocarditis and/or endocarditis accompanied by intracytoplasmic inclusion bodies in three of these animals. This raised the suspicion that the cardiac lesions might have a viral etiology. However, since most of the animals were found stranded and diseased on beaches, they had undergone some degree of autolysis before necropsies could be performed, and other logistic challenges also played a role, such as performing necropsies under field conditions where fresh-frozen tissues were not available, and, hence, attempts at virus isolation using tissue culture were precluded. While sea turtles can be persistently infected with herpes viruses [18,19], very little is known about the role of these viruses, if any, in cardiac inflammation. In humans and other animals, a wide range of viruses are involved in heart disease, either directly or indirectly, often leading to dilated cardiomyopathy [20]. Very little is currently known about the overall virome of GSTs [21], and nothing at all about the cardiac virome. Heidecker et al. [22] and Takeuchi et al. [23] recently investigated the virome of myocarditis in humans. While the former study did not identify any viruses in cardiac tissue by deep sequencing, Takeuchi et al. [23] detected multiple viruses, including the Epstein–Barr virus, human parvovirus B19, torque teno virus, and respiratory syncytial virus. However, like for many other virome studies, the pathophysiological role of these viruses could not be established.

Because only fixed tissues were available for nucleic acid extraction, genomic material was likely compromised. Moreover, enrichment for viruses during nucleic acid extraction was not performed, resulting in an overwhelming abundance of host and eDNA relative to viral reads in the sequence output. Nevertheless, a number of potentially interesting findings were made. Notably, several circovirus and CRESS virus genomes were identified, including BFDV-like sequences (Figure 4). Circoviruses are known to cause myocarditis in some other species, notably pigs, in which porcine circovirus 1–3 have all been implicated in acute and chronic myocarditis [24,25]. Attempts to detect the BFDV genome in the sea turtles by PCR gave ambiguous results, and immunohistochemical immunolabeling using a monoclonal antibody specific for BFVD [26] yielded no signal. However, this does not preclude the presence of a BFDV-like virus, as the PCR primers and the antibody may not have identical targets in the related sea turtle viruses. It was recently shown that BFDV occurs in non-psittacine avian species in which the virus does not cause any apparent disease [27]. We previously demonstrated that a poxvirus occurring in green sea turtles with skin lesions may have originated from an avian poxvirus [14]. Hence, some avian viruses may not be as host-specific as previously thought, and host-switching may lead to the evolution of new, related viruses in the new host.

It is acknowledged that sample contamination with avian tissue or fecal material may have occurred during either sampling, storage, or laboratory processing of the GST heart tissue. However, as not all samples had circovirus detected, it is unlikely that contamination occurred during laboratory processing.

While this study did not uncover a single likely viral etiology for cardiac inflammation in the GSTs, perhaps because we were restricted to assessing DNA viruses, the study has revealed that these animals are persistently infected with a wide range of viruses, some of which have the potential to affect the immune system (see, e.g., [24]) and, thereby, indirectly contribute to the development of myocarditis and endocarditis. It should also be kept in mind that the sea turtles are exposed to a range of other factors, including pollutants, high parasite loads, and malnutrition, as well as the substantial stress of nest-digging and egg-laying [28], which may interfere with immune defense mechanisms and allow viruses of low pathogenicity to replicate and incite inflammation in the heart. A prospective study with sampling and storage of tissues under more optimal conditions than was achievable in this study would allow a more comprehensive examination of both the RNA and DNA virome complemented with transcriptomic assessment of the immune response.

## Data Availability

All sequences analyzed have been deposited in NCBI GenBank under the accession numbers OQ992775- OQ992782, OQ980262- OQ980263, and OR198154- OR198166. Raw sequencing data from this study have been deposited in the NCBI Sequence Read Achieve (SRA) under the BioProject ID: PRJNA978835 (BioSample accessions: SAMN35717943–SAMN35717954; SRA accessions: SRR24940897–SRR24940908) (http://www.ncbi.nlm.nih.gov/sra/, accessed on 7 July 2023).

## Supplementary Materials

The following supporting information can be downloaded at https://www.mdpi.com/article/10.3390/v16071053/s1, Figure S1: Phylogenetic tree of beak and feather disease viruses; Table S1: Biodata, gross post mortem findings, and histological findings from green sea turtles that underwent post mortem histological examination from 2017 to 2020; Table S2: List of candidate viral genomes downloaded from NCBI; Table S3: Calculation of viral abundance.
