# Supplementary material for: DNA Virome in Cardiac Tissue from Green Sea Turtles (Chelonia mydas) with Myocarditis
Source: Viruses. 2024 Jun 29;16(7):1053. doi: 10.3390/v16071053 (PMC11281379; doi:10.3390/v16071053)
Supplement: Supplementary file 1 [file viruses-16-01053-s001.zip › Circovirus in Chelonia mydas - Supplementary Table S1 & Fig S1.pdf]

DNA virome in cardiac tissue from green sea turtles (*Chelonia mydas*) with myocarditis.

Christabel Hannon[1], Subir Sarker[2], Willy W. Suen[3], Helle Bielefeldt-Ohmann[4,5*]

Supplementary Table S1 & supplementary Figure S1

ID	NGS ID	Age Class	CCL (cm)	Wt. (kg)	BCS	Sex	PM Date	GPS	Euth.	Heart Pathology	Severity of Heart Pathology	TO Count	TA Count	Other Pathology	Summary
**A**		A	94.9	96.5	2	M	22.09.2017	-23.76620, 151.30492	Yes	- Endocarditis (LP-H)<br>- Myocarditis (LP-H)<br>- Ventricular thrombi<br>- Proximal aortic arteritis (H)<br>- Pericardial effusion.<br>- Intravascular thrombi.	Severe	0-1	0	- Poor nutritional status<br>- Coelomic effusion<br>- Gastroenteritis<br>- Systemic vasculitis (lymphohistiocytic, heterophilic)<br>- Splenic haemosiderosis	Inflammatory cardiovascular disease and transport stress.
**B**	**GT1**	A	98.2	83.4	4	F	03.11.2017	-23.76624, 151.30533	No	- Focal endocarditis (LH)<br>- Focal myocarditis (LH)<br>- Focal myocardial oedema.<br>- Aortic endarteritis	Mild, focal	0-1	0	- Pulmonary endarteritis	Inflammatory cardiovascular disease and transport stress.
**C**	**GT2**	A	93.0	104.5	3	F	04.11.2017	-23.76624, 151.30533	No	- Endocarditis (E, LH)<br>- Myocarditis (LH)<br>- Pericarditis<br>- Proximal aortic subendothelial arteritis (E, H)	Severe	0-1	0	- Systemic vasculitis<br>- Pancreatitis<br>- Cholangiohepatitis<br>- Cystitis<br>- Nephritis<br>- Meningitis (L)<br>- Peumonitis (LH)<br>- Renal calcium oxlaosis<br>- Poxvirus skin infection	Multi-organ inflammatory disease of unknown origin and transport stress.
**D**	**GT3**	SA	75.8	51.6	4	M	04.11.2017	-23.76624, 151.30533	No	- Endocarditis (H)<br>- Myocarditis (H)<br>- Myocardial oedema<br>- Myocardial fibrosis	Moderate	0-1	0	- Nephritis (LH)	Inflammatory cardiovascular disease, nephritis and transport stress.
**E**	**GT7**	A	113.3	134.4	3	F	27.06.2018	-23.94775, 151.35948	Yes	- Endocarditis (mononuclear)	Mild	0	0	- Systemic vasculitis.<br>- Cystitis<br>- TMJ articular disc displacement.<br>- Hepatic neoplasia.	Unknown.<br><br>DDx: neoplastic hepatopathy, viral cardiovascular disease, TMJ pain.
**F**	**GT5**	J	38.8	6.4	4	M	29.09.2018	-23.83507, 151.25264*	No	- Endocarditis (H)<br>- Myocarditis (LH)	Moderate	0-2	0	- Systemic vasculitis<br>- *Plesiochorus cymbiformis* bladder infection<br>- Cystitis<br>- Gastroenteritis<br>- Fungal pneumonia<br>- Splenic lymphoid depletion	Inflammatory cardiovascular disease, immunosuppression and secondary infections.
**G**	**GT6**	J	43.9	9.6	3	F	30.09.2018	-23.93735, 151.35433	No	- Endocarditis (L)<br>- Myocarditis (LP)<br>- Trematode adults in ventricular lumen.	Mild	0-5	0-3	- Systemic vasculitis<br>- *Plesiochorus cymbiformis* bladder infection<br>- Cystitis<br>- Nephritis<br>- Terminal emphysema	Inflammatory cardiovascular disease, immunosuppresion and secondary infections.

														- Focal fungal pneumonia<br>- Splenic lymphoid depletion<br>- Gastroenteritis	
**H**	**GT4**	J	46.9	N/A	1	F	02.10.2018	-23.76074, 151.30923	No	- Nil	Nil	0-2	0	- Poor nutritional status<br>- Coelomic effusion<br>- Bacterial perivascular hepatitis<br>- Systemic intravascular thrombosis.<br>- Pulmonary vasculopathy<br>- Meningitis	Peracute hepatitis secondary to sepsis.
**I**	**GT9**	J	45.8	8.5	1	M	20.05.2019	-23.85593, 151.31583	Yes	- Pericardial effusion<br>- Endocarditis (LH)<br>- Myocarditis (LH)	Severe	0-2	0	- Poor nutritional status<br>- MSK predation wounds<br>- Systemic vasculitis<br>- Hepatic granulomas<br>- Bacterial pneumonia	Fatal predation wounds secondary to debilitation from inflammatory cardiovascular disease, bacterial pulmonary infection and hepatic granulomas of unknown aetiology.
**J**	**GT12**	A	109.<br>1	131	4	F	22.05.2019	-23.15404, 150.76857*	No	- Endocarditis (LH)<br>- Myocarditis (LH)	Mild	0-3	0-1	- Post-ovulatory dystocia<br>- Oviduct torsion and necrosis<br>- Interstitial nephritis<br>- Systemic vasculitis<br>- Acute<br>- Cystitis<br>- DIC<br>- Enteritis with acute necrosis and ulceration	Septicaemia and DIC secondary to post-ovulatory dystocia.
**K**	**GT11**	J	43.5	6.4	1	F	26.05.2019	-23.92930, 151.35703	Yes	- Pericardial effusion<br>- Endocarditis (LH)<br>- Myocarditis (LH with bacteria)<br>- Trematode adults in ventricular lumen.	Moderate*	10-50	2-5	- Poor nutritional status<br>- Cachexia<br>- Dehydration<br>- Systemic spirorchiid-associated granulomatous inflammation.<br>- Bone marrow atrophy<br>- Cortical bone thinning	Severe pathology secondary to spirochiid infection.
**L**	**GT10**	J	50.0	N/A	4	F	11.07.2019	-23.93392, 151.35434	No	- Endocarditis (LH)<br>- Myocarditis (LH)<br>- Aortic endarteritis<br>- Aortic thrombosis (non-patent)	Severe	0-6	0-3	- Fibropapillomatosis<br>- Nasal cavity occlusion<br>- Glomerulonephritis<br>- Pneumonitis<br>- Cystitis<br>- Gastritis<br>- Hepatitis	Multi-organ inflammatory disease of unknown origin
**M**	**GT8**	J	45.1	5.6	1	M	29.09.2019	-23. 76240, 151.33141	Yes	- Endocarditis (LH)<br>- Myocarditis (L)<br>- Pericarditis<br>- Aortic endarteritis and abscessation	Severe	0-8	0	- Poor nutritional status<br>- Systemic vasculitis<br>- *Plesiochorus cymbiformis* bladder infection<br>- Cystitis	Unknown<br><br>DDx: spirochiid infection, multi-systemic viral

													- Cholangiohepatitis<br>- Gastroenteritis<br>- Intravascular adult spirorchiid flukes.	infection, gastrointestinal malabsorption.
**N**	J	50.5	11.4	2	M	22.09.2020	-23.91906, 151.34521	No	- Endocarditis (LH)<br>- Myocarditis (L)	Mild**	0-1	0	- Poor nutritional status<br>- Severe *Ozobranchus* spp. ectoparasitism<br>- Plastron trauma<br>- Focal ulcerative stomatitis<br>- Oral mucosal ulceration<br>- Diffuse severe necrotising gastroenteritis with bacterial infection.<br>- Benign testicular cyst<br>- Packed sand in larynx<br>- Systemic vasculitis	Severe necrotising gastroenteritis and endotoxaemia.
**O**	J	48.4	9.2	2	M	23.09.2020	-23.80878, 151.26817	No	- Endocarditis (L)<br>- Myocarditis (L)<br>- Aortic vasculitis<br>- Aortic spirorchiid granuloma<br>- Adult flukes in atrium.	Mild*	0-5	0-2	- Poor nutritional status<br>- Intravascular adult spirorchiid flukes.<br>- Necrotic oesophagitis<br>- Gastroenteritis	Severe necrotising oesophagitis and moderate to severe spirorchiid infection.
**P**	SA	66.0	26.0	3	F	05.11.2020	-23.90090, 151.33249	No	- Aortic aneurysm<br>- Peritonitis<br>- Endocarditis<br>- Myocarditis<br>- Pericarditis<br>- Pericardial tear<br>- Aortic spirorchiid granuloma<br>- Adult flukes in atrium.<br>- Intraluminal bacteria.	Moderate**	0-3	0-3	- Increased *Ozobranchus* spp. Ectoparasitism<br>- Colonic volvulus<br>- Systemic vascular thrombosis<br>- Intestinal perforation	Strangulating colonic volvulus with acute septicaemia.

Table S1: summary of biodata, gross post-mortem findings and histological findings from green sea turtles that underwent *post-mortem* histological examination from 2017-2020.

J = juvenile, SA = subadult, A = adult, CCL = curved carapace length, Wt. = weight, BCS = body condition score, F = female, M = male, L = lymphocytic, H = histiocytic, LH = lymphohistiocytic, LP = lymphoplasmacytic, E = eosinophilic, TO = trematode ova in cardiac tissue, TA = trematode adults in cardiac tissue.

Euth. represents whether the turtle was humanely euthanased by a registered veterinarian, or whether it died spontaneously of natural causes.

NGS ID represents the identification assigned to sample during NGS processing.

Dates presented as DD/MM/YYYY.

GPS presented as latitude, longitude co-ordinates.

Trematode ova and adult count presented as a range per 10x objective field.

*Heart disease present but histological lesions associated with spirorchiid infection, rather than a suspected viral aetiology.

**Heart disease present but histological lesions associated with sepsis, rather than a suspected viral aetiology.

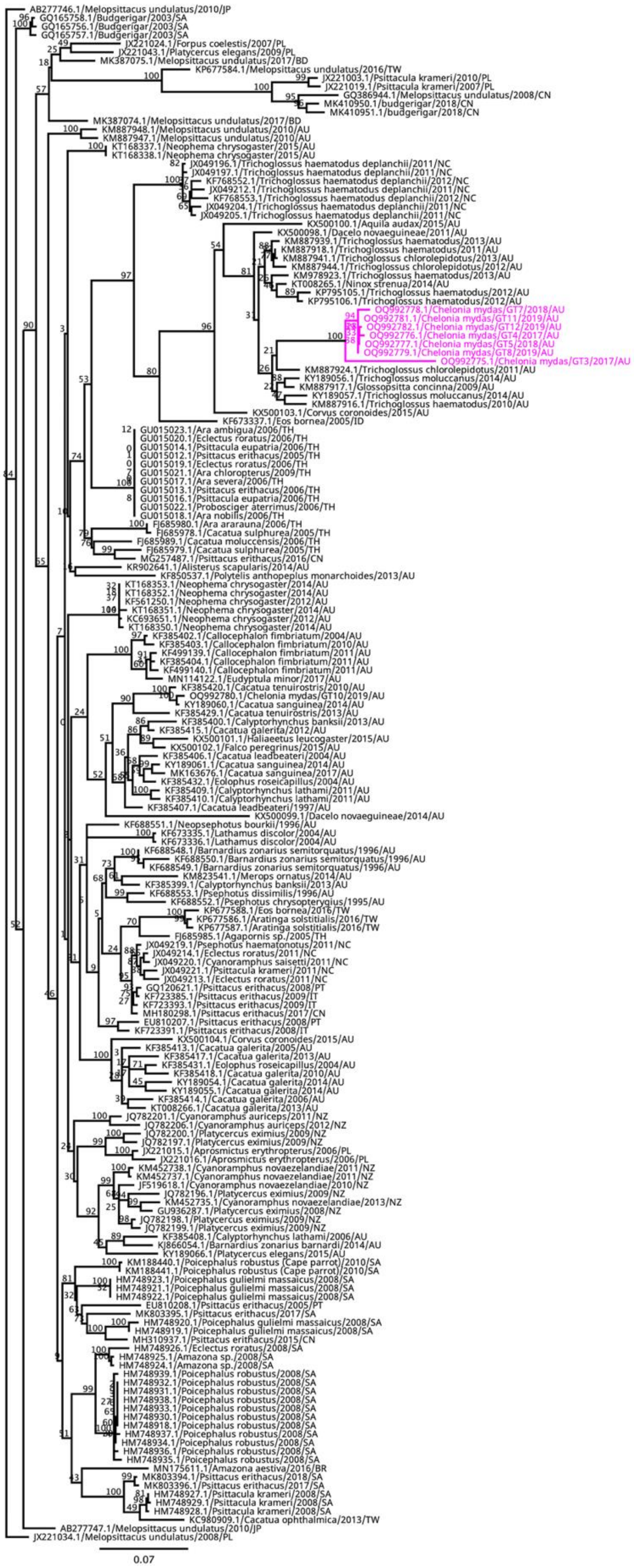

Supplementary Figure S1. Phylogenetic tree of beak and feather disease virus (BFDV) with placement of the BFDV detected in the green sea turtles of this study. The numbers on the left show bootstrap values as percentages, and the labels at branch tips refer to GenBank accession number/sampling host/year/country code. The sequences correspondence to the BFDV detected in this study is shown in pink colour.
